# Genetically Predicted Milk Intake and Risk of Neurodegenerative Diseases

**DOI:** 10.3390/nu13082893

**Published:** 2021-08-23

**Authors:** Zhizhong Zhang, Mengmeng Wang, Shuai Yuan, Susanna C. Larsson, Xinfeng Liu

**Affiliations:** 1 Department of Neurology, Jinling Hospital, Medical School of Nanjing University, Nanjing 210002, China; zhizhongn@163.com; 2 Department of Neurology, The First People’s Hospital of Changzhou, The Third Affiliated Hospital of Soochow University, Changzhou 213004, China; w935017050@163.com; 3 Unit of Cardiovascular and Nutritional Epidemiology, Institute of Environmental Medicine, Karolinska Institutet, SE-171 77 Stockholm, Sweden; shuai.yuan@ki.se (S.Y.); susanna.larsson@ki.se (S.C.L.)

**Keywords:** milk, causal effect, neurodegenerative disease

## Abstract

Milk intake has been associated with risk of neurodegenerative diseases in observational studies. Nevertheless, whether the association is causal remains unknown. We adopted Mendelian randomization design to evaluate the potential causal association between milk intake and common neurodegenerative diseases, including multiple sclerosis (MS), Alzheimer’s disease (AD), amyotrophic lateral sclerosis (ALS), and Parkinson’s disease (PD). Genetic associations for neurodegenerative diseases were obtained from the International Multiple Sclerosis Genetics Consortium (*n* = 80,094), FinnGen consortium (*n* = 176,899), AD GWAS (*n* = 63,926), Web-Based Study of Parkinson’s Disease (*n* = 308,518), PDGene (*n* = 108,990), and ALS GWAS (*n* = 80,610). Lactase persistence variant rs4988235 (*LCT*-13910 C > T) was used as the instrumental variable for milk intake. Genetically predicted higher milk intake was associated with a decreased risk of MS and AD and with an increased risk of PD. For each additional milk intake increasing allele, the odds ratios were 0.94 (95% confidence intervals [CI]: 0.91–0.97; *p* = 1.51 × 10^−4^) for MS, 0.97 (0.94–0.99; *p* = 0.019) for AD and 1.09 (95%CI: 1.06–1.12, *p* = 9.30 × 10^−9^) for PD. Genetically predicted milk intake was not associated with ALS (odds ratio: 0.97, 95%CI: 0.94–1.01, *p* = 0.135). Our results suggest that genetically predicted milk intake is associated with a decreased risk of MS and AD but with an increased risk of PD. Further investigations are needed to clarify the underlying mechanisms.

## 1. Introduction

Neurodegenerative diseases lead to severe social and economic burdens in aging populations [1]. The causes of neurodegenerative diseases are not yet well understood. Dietary factors play a key role in the development of neurodegenerative diseases, among which milk intake has received considerable interest. Milk is a rich source of essential nutrients and a variety of anabolic hormones, which can nourish and facilitate growth [2].

The effects of milk intake on the risk of neurodegenerative diseases have been widely investigated. Observational studies have shown that milk intake is associated with a reduced risk of multiple sclerosis (MS) [3,4] and Alzheimer’s disease (AD) [5,6], but with an increased risk of Parkinson’s disease (PD) [5,7,8,9]. The association between milk intake and amyotrophic lateral sclerosis (ALS) is largely unknown.

Since observational studies are prone to potential biases, especially reverse causality and confounding, the causal effect of milk intake on neurodegenerative diseases risk is unknown. The Mendelian randomization (MR) design adopts genetic variants as the instrumental variable for an exposure to reduce these potential biases, which could enhance the causal inference [10].

**Figure 1:**
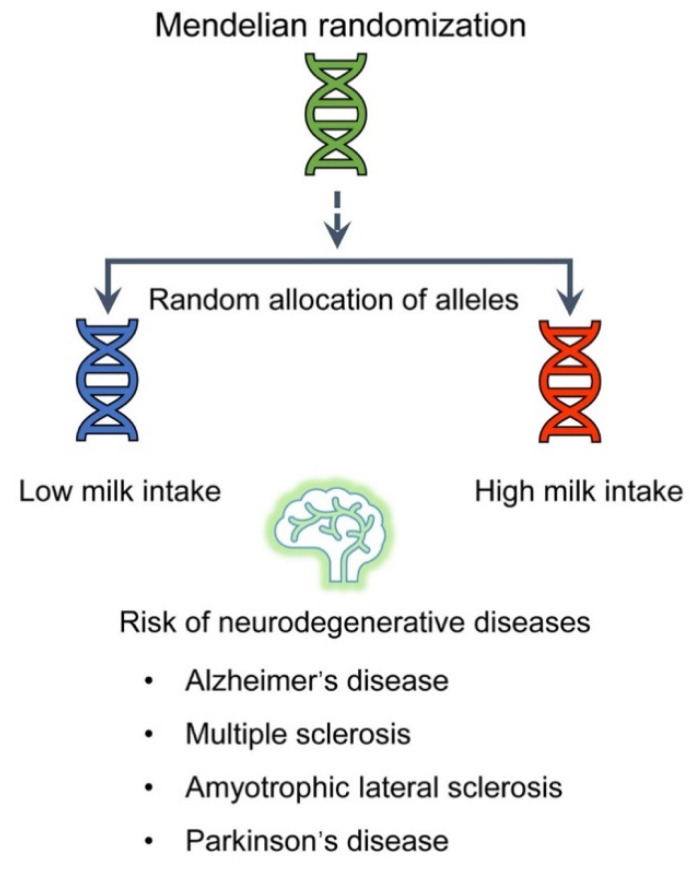
Overview of this MR study.

The *lactase* (*LCT*) gene encodes lactase, which metabolizes milk sugar. The genetic variant rs4988235, near the *LCT* gene (*LCT*-13910 C > T), is related to lactase persistence (LP) and increased milk intake among Europeans [11,12]. Here, we evaluated the causal association of milk intake with neurodegenerative diseases (MS, AD, ALS, and PD) by adopting the genetic variant near *LCT* as the instrumental variable for milk intake (Figure 1).

## 2. Methods

### 2.1. Study Design

This study is based on the MR design, which relies on three main assumptions. First, the instrumental variable should affect the exposure. Second, the instrumental variable should not be related to confounders. Third, the instrumental variable should affect the outcome only through the exposure.

### 2.2. Outcome Data Sources

Summary-level data for MS were obtained from the International Multiple Sclerosis Genetics Consortium (IMSGC, MS genome-wide association study (GWAS): 14,802 cases and 26,703 controls [13]; MS Immunochip: 14,498 cases and 24,091 controls, Table 1) [14]. Genetic results for AD were obtained from the the International Genomics of Alzheimer’s Project (IGAP, 21,982 cases and 41,944 controls) [15] and FinnGen consortium R4 release (3,060 cases and 173,839 controls). Genetic association estimates for ALS were retrieved from a recent large GWAS of ALS (20,806 cases and 59,804 controls) [16]. Summary-level data for PD were obtained from the Web-Based Study of Parkinson’s Disease (PDWBS, 6,476 cases 302,042 controls) [17] and PDGene (13,708 cases, 95,282 controls) [18]. In our study, the analysis was restricted to European subjects to minimize population stratification bias. Details of case ascertainment and genotyping for these neurodegenerative diseases have been described in the original studies.

### 2.3. Genetic Instrument

**Figure 2:**
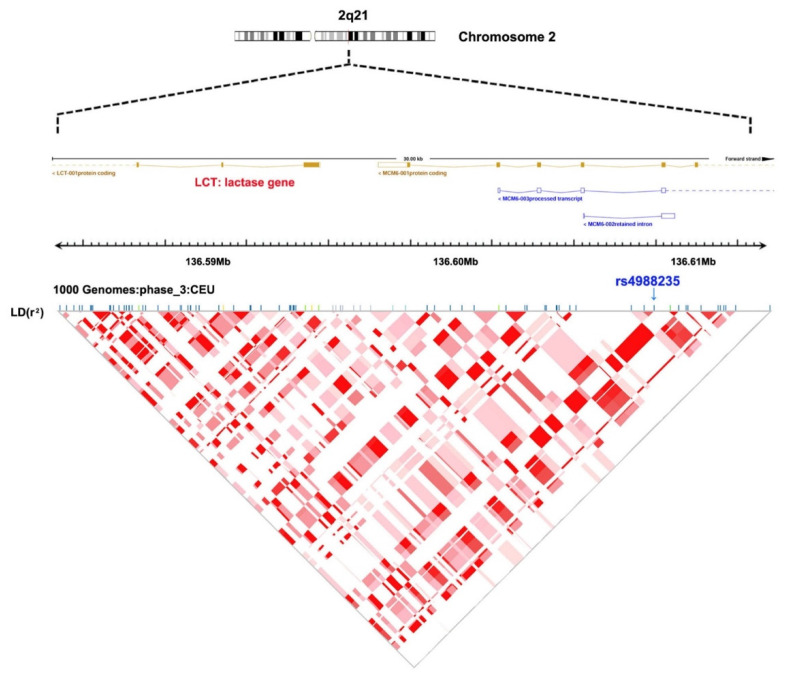
Rs4988235 on chromosome 2q21 and linkage disequilibrium pattern of single-nucleotide polymorphisms based on 1000 genomes (phase_3, CEU).

We adopted rs4988235 as the genetic instrument for milk intake, which is located 13,910 bp upstream from the *LCT* gene (Figure 2). This genetic variant has been shown to be strongly associated with milk intake in Europeans [11,12], thereby fulfilling the first MR assumption. In the EPIC-InterAct study, the median milk intake was 162 g/day, and each additional lactase persistence allele (T) of rs4988235 was associated with 17.1 g/day (95% confidence interval [CI]: 10.6–23.6, *p* = 2 × 10^−7^) higher milk intake [12]. In a Danish cohort comprising 73,715 participants, milk intake increased by 0.58 (95% CI: 0.49–0.68; *p* = 9 × 10^−36^) glasses/week per an additional T-allele of rs4988235 [11]. The genetic variant associated with milk intake (rs4988235) explained 2% of the variance in milk intake and had an F statistic of 515 [11].

### 2.4. Statistical Analysis

The ratio estimate for rs4988235 was calculated by dividing the beta-coefficient of outcome by the beta-coefficient of milk intake. Odds ratios (ORs) and corresponding 95% CIs were computed per additional T-allele of rs4988235. For outcomes with two independent samples, the MR estimates were combined with a fixed-effects meta-analysis. We evaluated the heterogeneity between studies with the *I*^2^ statistics. The analyses were performed using Stata 14.0.

## 3. Results

### 3.1. Basic Characteristics

Studies and datasets adopted in the present MR analyses were shown in Table 1. The outcome data sources included four neurodegenerative diseases (MS, AD, ALS, and PD). All analyses in the present MR study were based on individuals of European ancestry (Table 1).

The associations of the T allele of rs4988235 with neurodegenerative diseases are shown in Table 2. Summary-level data (regression coefficient and standard error) for rs4988235 were available in all outcome databases (Table 2).

### 3.2. Genetically Predicted Milk Intake and Risk of Neurodegenerative Diseases

**Figure 3:**
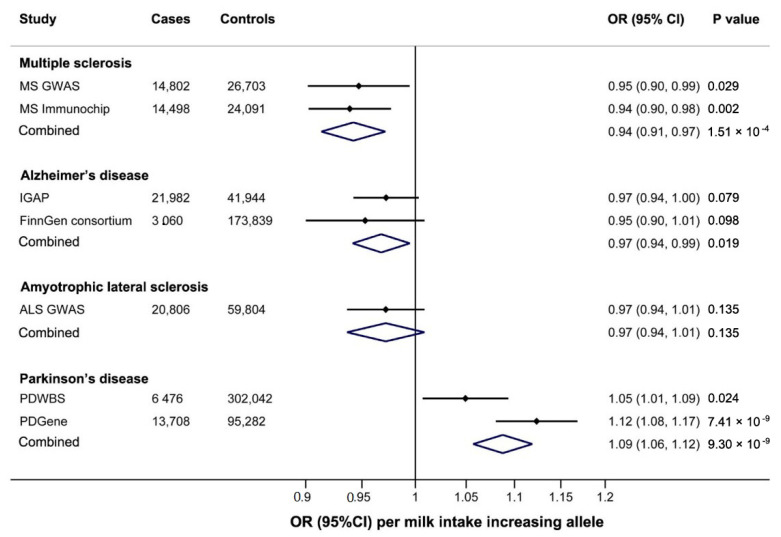
Association of genetically predicted higher milk intake with neurodegenerative diseases. CI: confidence interval, OR: odds ratio, IGAP: International Genomics of Alzheimer’s Project, PDWBS: Web-Based Study of Parkinson’s Disease.

Genetically predicted milk intake was associated with a decreased risk of MS in both the MS GWAS (OR: 0.95, 95%CI: 0.90–0.99) and MS Immunochip (OR: 0.94, 95%CI: 0.90–0.98). In the combined analysis, the OR and 95% CI for each additional milk consumption increasing allele (T) was 0.94 (0.91–0.97; *p* = 1.51 × 10^−4^, Figure 3) without heterogeneity between studies (*p* = 0.788, *I*^2^ = 0%).

Genetically predicted milk intake was also inversely associated with AD risk (Figure 3). In the combined analysis of IGAP and FinnGen consortium, the OR for each additional milk intake-increasing allele (T) was 0.97 (0.94–0.99, *p* = 0.019, Figure 3) without heterogeneity between studies (*p* = 0.545, *I*^2^ = 0%). Genetically predicted milk intake was not associated with ALS (OR: 0.97; 95% CI: 0.94–1.01; *p* = 0.135, Figure 3).

Genetically predicted milk intake was associated with an increased risk of PD in both PDWBS (OR: 1.05, 95%CI: 1.01–1.09) and PDGene (OR: 1.12, 95%CI: 1.08–1.17). In the combined analysis of PDWBS and PDGene, the OR and 95% CI for each additional milk intake-increasing allele (T) was 1.09 (1.06–1.12; *p* = 9.3 × 10^−9^, Figure 3).

## 4. Discussion

In the present study, we investigated the causal relationship between genetically predicted milk intake and the risk of four common neurodegenerative diseases using the MR design. To the best of our knowledge, this is the first study using the MR method to explore the causal association between milk intake and risk of neurodegenerative diseases.

Milk is widely consumed worldwide and is a crucial modifiable risk factor for neurodegenerative diseases. In recent years, increasing attention has been paid to the relationship between milk intake and risk of neurodegenerative diseases. Milk is rich in nutrients such as protein, fat, vitamins, and calcium [19]. Our MR study showed that genetically predicted higher milk intake was associated with a decreased risk of MS and AD but with an increased risk of PD, consistent with results of observational studies [3,4,5,6].

A previous case-control study (660 patients and 421 controls) showed that milk intake was associated with decreased risk of MS [3]. Another case-control study (536 patients and 399 controls) indicated that dairy product consumption was significantly lower in MS patients (56.6%) than in control participants (67.5%, *p* = 0.01) [4]. For AD, results of a meta-analysis indicated that elevated milk intake was associated with a lower AD risk (OR = 0.63, 95%CI: 0.44–0.90) [6].

The protective effect of milk intake on MS and AD might be partially mediated through vitamin D and calcium. Vitamin D regulates calcium and phosphate metabolism. An association between low vitamin D levels and increased risk of MS was observed [20]. In a prospective nested case-control study (148 MS cases and 296 controls), the risk of MS was reduced by 41% for a 50-nmol/L increase in 25-hydroxyvitamin D [21]. MR studies have also indicated that circulating vitamin D levels are inversely associated with MS risk [22,23]. Furthermore, in the prospective Rotterdam study, lower serum vitamin D levels were associated with an increased risk of AD (hazard ratio: 1.13; 95% CI: 1.03–1.24) [24].

Calcium is essential for maintaining physiological processes in the cells [25]. Dysregulation of calcium homeostasis has been associated with MS and AD development [25,26]. A case-control study showed that the frequency of calcium supplementation among the control population (30.4%) was significantly higher than in MS patients (14.9%, *p* < 0.001), and calcium supplementation was associated with decreased risk of MS (OR = 0.44, 95%CI: 0.27–0.71) [3]. Furthermore, higher genetically predicted serum calcium has been shown to be associated with a lower risk of AD (OR = 0.57, 95% CI: 0.35–0.95) [27]. Thus, milk could have a protective effect against MS and AD through vitamin D and calcium.

Our results indicate that higher milk intake is associated with an increased risk of PD. This finding is consistent with results of observational studies [5,7,8,9]. A previous study indicated that milk could increase risk of PD by decreasing the serum uric acid level [28]. Many studies have demonstrated that urate has a protective effect against PD, and increasing plasma urate might decrease PD risk and postpone PD progression [29,30].

A limitation is that potential non-linear relationships between milk intake and risk of neurodegenerative diseases could not be evaluated in the present study due to the summary-level data used in our MR analyses. Secondly, we could not assess potential mechanisms behind the observed associations between milk intake and neurodegenerative diseases. Thirdly, whether our results could be applied to other forms of dairy products needs to be investigated in future. Finally, we cannot exclude that the used genetic instrument for milk intake affects the risk of neurodegenerative diseases through other pathways than via milk intake, thereby violating the second and third MR assumptions. For example, an increased intake of milk is likely to lead to reduced intake of other foods (to maintain energy balance) that might be associated with risk of neurodegenerative diseases. However, to date, no other food item has been established to affect the risk of the studied neurodegenerative diseases.

## 5. Conclusions

This MR study found that genetically predicted milk intake might be associated with a decreased risk of MS and AD but with an increased risk of PD. Further investigations are needed to clarify the potential underlying mechanisms.

## Figures and Tables

**Table 1:** Studies and datasets adopted in the present analyses.

Outcome	Consortium	Population	Cases	Controls
Multiple sclerosis	International Multiple Sclerosis Genetics Consortium-MS GWAS	European	14,802	26,703
	International Multiple Sclerosis Genetics Consortium-MS Immunochip	European	14,498	24,091
Alzheimer’s disease	International Genomics of Alzheimer’s Project	European	21,982	41,944
	FinnGen consortium	European	3,060	173,839
Parkinson’s disease	Web-Based Study of Parkinson’s Disease	European	6,476	302,042
	PDGene	European	13,708	95,282
Amyotrophic lateral sclerosis	Project MinE, the database of Genotypes and Phenotypes, the HYPERGENES Project, the Wellcome Trust Case Control Consortium, National Institute on Aging	European	20,806	59,804

**Table 2:** Association between rs4988235 variant T allele and neurodegenerative diseases. Beta, regression coefficient; SE, standard error.

Disease	Database	Beta	SE	*p*
Multiple sclerosis	MS GWAS	−0.054	0.025	2.93 × 10^−2^
	MS Immunochip	−0.063	0.020	1.97 × 10^−3^
Alzheimer’s disease	IGAP	−0.028	0.016	7.88 × 10^−2^
	FinnGen consortium	−0.048	0.029	9.76 × 10^−2^
Parkinson’s disease	PDWBS	0.048	0.021	2.37 × 10^−2^
	PDGene	0.117	0.020	7.41 × 10^−9^
Amyotrophic lateral sclerosis	ALS GWAS	−0.028	0.019	1.35 × 10^−1^

## Data Availability

The data used to conduct the analyses in present study were obtained from public GWASs summary statistics (please see Table 1 and Table 2).
